# 1,5-Bis(4-bromo­phen­yl)-3-phenyl­pentane-1,5-dione

**DOI:** 10.1107/S1600536808023970

**Published:** 2008-08-06

**Authors:** Kao-Zhen Li, Yu-Ting Chen, Chuan-Wen Zhao, Guo-Dong Wei, Qing-Peng He

**Affiliations:** a College of Chemistry and Chemical Engineering, Liaocheng University, Shandong 252059, People’s Republic of China; b Department of Chemsitry, Dezhou University, Dezhou 100835, People’s Republic of China; c Bureau of Quality and Technical Supervision, Liaocheng 252000, Shandong, People’s Republic of China; d Shandong Donge Experimental High School, Donge, Shandong Province 252200, People’s Republic of China

## Abstract

The asymmetric unit of the title compound, C_23_H_18_Br_2_O_2_, contains two independent mol­ecules with slightly different conformations. In the absence of classical inter­molecular inter­actions, the crystal packing is stabilized by van der Waals forces.

## Related literature

For the crystal structures of two related 1,5-diketones, see: Das *et al.* (1994 ▶); Huang *et al.* (2006 ▶).
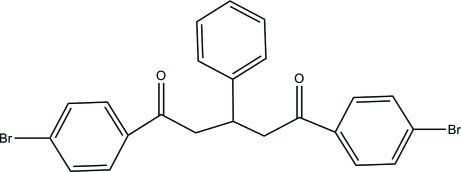


## Experimental

### 

#### Crystal data

C_23_H_18_Br_2_O_2_
*M* *_r_* = 486.19
Monoclinic, 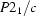

*a* = 26.460 (3) Å
*b* = 6.2080 (17) Å
*c* = 26.320 (3) Å
β = 112.020 (2)°
*V* = 4008.0 (12) Å^3^
*Z* = 8
Mo *K*α radiation
μ = 4.06 mm^−1^
*T* = 298 (2) K
0.40 × 0.35 × 0.18 mm

#### Data collection

Siemens SMART CCD area-detector diffractometer
Absorption correction: multi-scan (*SADABS*; Sheldrick, 1996 ▶) *T*_min_ = 0.293, *T*_max_ = 0.529 (expected range = 0.267–0.482)
18598 measured reflections
6974 independent reflections
3492 reflections with *I* > 2σ(*I*)
*R*_int_ = 0.114

#### Refinement

*R*[*F* ^2^ > 2σ(*F* ^2^)] = 0.099
*wR*(*F* ^2^) = 0.301
*S* = 0.93
6974 reflections
487 parameters
H-atom parameters constrained
Δρ_max_ = 0.98 e Å^−3^
Δρ_min_ = −1.01 e Å^−3^

### 

Data collection: *SMART* (Siemens, 1996 ▶); cell refinement: *SAINT* (Siemens, 1996 ▶); data reduction: *SAINT*; program(s) used to solve structure: *SHELXS97* (Sheldrick, 2008 ▶); program(s) used to refine structure: *SHELXL97* (Sheldrick, 2008 ▶); molecular graphics: *SHELXTL* (Sheldrick, 2008 ▶); software used to prepare material for publication: *SHELXTL*.

## Supplementary Material

Crystal structure: contains datablocks I, global. DOI: 10.1107/S1600536808023970/cv2434sup1.cif

Structure factors: contains datablocks I. DOI: 10.1107/S1600536808023970/cv2434Isup2.hkl

Additional supplementary materials:  crystallographic information; 3D view; checkCIF report

## Figures and Tables

**Fig. 1.**
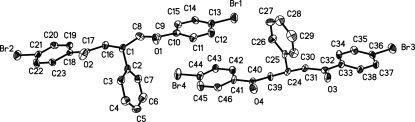
Two independent molecules (I) with the atom numbering scheme and 30% probability displacement ellipsoids. Hydrogen atoms are omitted for clarity.

## supplementary crystallographic information

### Comment

In continuation of our ongoing program directed to the development of environmentally benign methods of chemical synthesis, we describe in this paper a user-friendly, solvent-free protocol for the synthesis of 1,5-diketones starting from the fragrant aldehydes and fragrant ketones in the presence of NaOH under solvent-free conditions. Using this method, which can be considered as a a general method for the synthesis of 1,5-diketones, we obtained the title compound, (I). We present here its crystal structure.

In the molecule (Fig. 1), the bond lengths and angles (Table 1) are normal and correspond to those observed in 1,3,5-triphenyl-pentane-1,5-diketone (Das *et al.*, 1994), 1,5-Diphenyl-3-(2-pyridyl)pentane-1,5-dione (Huang *et al.*, 2006)

### Experimental

4-Bromoacetophenone (10 mmol), freshly distilled benzaldehyde (5 mmol) and NaOH (10 mmol) were aggregated with glass paddle in an open flask. The resulting mixture was washed with water for several times for removing NaOH, and recrystalized from ethanol, and afforded the title compound as a crystalline solid. C_23_H_18_Br_2_O_2_: C 56.82, H 3.73%; Found: C 56.85, H 3.62%.

### Refinement

All H atoms were positioned geometrically and refined using a riding model, with C—H = 0.93–0.98 Å and *U*_iso_(H) = 1.2 *U*_eq_(C).

### Crystal data

**Table:**

C_23_H_18_Br_2_O_2_	*F*_000_ = 1936
*M**_r_* = 486.19	*D*_x_ = 1.611 Mg m^−^^3^
Monoclinic, *P*2_1_/*c*	Mo *K*α radiation λ = 0.71073 Å
*a* = 26.460 (3) Å	Cell parameters from 3565 reflections
*b* = 6.2080 (17) Å	θ = 2.5–24.0º
*c* = 26.320 (3) Å	µ = 4.06 mm^−^^1^
β = 112.020 (2)º	*T* = 298 (2) K
*V* = 4008.0 (12) Å^3^	Needle, colourless
*Z* = 8	0.40 × 0.35 × 0.18 mm

### Data collection

**Table:**

Siemens SMART CCD area-detector diffractometer	6974 independent reflections
Radiation source: fine-focus sealed tube	3492 reflections with *I* > 2σ(*I*)
Monochromator: graphite	*R*_int_ = 0.114
*T* = 298(2) K	θ_max_ = 25.0º
φ and ω scans	θ_min_ = 1.6º
Absorption correction: multi-scan(SADABS; Sheldrick, 1996)	*h* = −31→31
*T*_min_ = 0.294, *T*_max_ = 0.529	*k* = −7→7
18598 measured reflections	*l* = −31→25

### Refinement

**Table:**

Refinement on *F*^2^	Secondary atom site location: difference Fourier map
Least-squares matrix: full	Hydrogen site location: inferred from neighbouring sites
*R*[*F*^2^ > 2σ(*F*^2^)] = 0.099	H-atom parameters constrained
*wR*(*F*^2^) = 0.301	*w* = 1/[σ^2^(*F*_o_^2^) + (0.1338*P*)^2^ + 45.7826*P*] where *P* = (*F*_o_^2^ + 2*F*_c_^2^)/3
*S* = 0.93	(Δ/σ)_max_ = 0.003
6974 reflections	Δρ_max_ = 0.98 e Å^−^^3^
487 parameters	Δρ_min_ = −1.01 e Å^−^^3^
Primary atom site location: structure-invariant direct methods	Extinction correction: none

### Special details

**Table:**

Geometry. All e.s.d.'s (except the e.s.d. in the dihedral angle between two l.s. planes) are estimated using the full covariance matrix. The cell e.s.d.'s are taken into account individually in the estimation of e.s.d.'s in distances, angles and torsion angles; correlations between e.s.d.'s in cell parameters are only used when they are defined by crystal symmetry. An approximate (isotropic) treatment of cell e.s.d.'s is used for estimating e.s.d.'s involving l.s. planes.
Refinement. Refinement of *F*^2^ against ALL reflections. The weighted *R*-factor *wR* and goodness of fit *S* are based on *F*^2^, conventional *R*-factors *R* are based on *F*, with *F* set to zero for negative *F*^2^. The threshold expression of *F*^2^ > σ(*F*^2^) is used only for calculating *R*-factors(gt) *etc*. and is not relevant to the choice of reflections for refinement. *R*-factors based on *F*^2^ are statistically about twice as large as those based on *F*, and *R*- factors based on ALL data will be even larger.

### Fractional atomic coordinates and isotropic or equivalent isotropic displacement parameters (Å^2^)

**Table:**

	*x*	*y*	*z*	*U*_iso_*/*U*_eq_	
Br1	0.73025 (7)	0.3535 (3)	0.61892 (7)	0.0740 (6)	
Br2	0.37401 (6)	0.3627 (3)	0.99418 (7)	0.0764 (6)	
Br3	1.11853 (7)	0.2623 (3)	0.50238 (7)	0.0784 (6)	
Br4	0.74151 (7)	0.2505 (3)	0.84625 (8)	0.0806 (6)	
O1	0.6129 (4)	1.0109 (15)	0.7515 (4)	0.057 (3)	
O2	0.5077 (4)	0.9997 (15)	0.8761 (5)	0.066 (3)	
O3	1.0173 (4)	0.9354 (15)	0.6476 (4)	0.054 (2)	
O4	0.9006 (4)	0.9293 (17)	0.7644 (4)	0.061 (3)	
C1	0.5691 (4)	0.8137 (19)	0.8217 (5)	0.042 (3)	
H1	0.5509	0.9501	0.8068	0.050*	
C2	0.6216 (5)	0.8633 (19)	0.8677 (5)	0.039 (3)	
C3	0.6304 (5)	1.060 (2)	0.8947 (6)	0.051 (3)	
H3	0.6032	1.1641	0.8831	0.062*	
C4	0.6795 (6)	1.106 (2)	0.9395 (6)	0.062 (4)	
H4	0.6842	1.2385	0.9572	0.074*	
C5	0.7200 (6)	0.954 (3)	0.9566 (6)	0.062 (4)	
H5	0.7524	0.9827	0.9859	0.074*	
C6	0.7125 (6)	0.760 (3)	0.9303 (7)	0.063 (4)	
H6	0.7406	0.6597	0.9412	0.076*	
C7	0.6646 (5)	0.709 (2)	0.8883 (5)	0.046 (3)	
H7	0.6600	0.5724	0.8730	0.056*	
C8	0.5795 (6)	0.690 (2)	0.7748 (5)	0.051 (3)	
H8A	0.5972	0.5545	0.7891	0.061*	
H8B	0.5447	0.6586	0.7459	0.061*	
C9	0.6137 (5)	0.811 (2)	0.7511 (5)	0.041 (3)	
C10	0.6433 (5)	0.6958 (19)	0.7214 (5)	0.042 (3)	
C11	0.6838 (5)	0.806 (2)	0.7098 (5)	0.046 (3)	
H11	0.6935	0.9453	0.7227	0.056*	
C12	0.7098 (5)	0.703 (2)	0.6782 (6)	0.055 (4)	
H12	0.7370	0.7732	0.6702	0.065*	
C13	0.6944 (5)	0.495 (2)	0.6594 (5)	0.045 (3)	
C14	0.6569 (5)	0.382 (2)	0.6730 (5)	0.047 (3)	
H14	0.6493	0.2386	0.6624	0.056*	
C15	0.6303 (5)	0.4833 (18)	0.7029 (5)	0.041 (3)	
H15	0.6035	0.4095	0.7109	0.049*	
C16	0.5308 (5)	0.6795 (19)	0.8409 (5)	0.042 (3)	
H16A	0.5016	0.6213	0.8091	0.051*	
H16B	0.5509	0.5594	0.8628	0.051*	
C17	0.5065 (4)	0.814 (2)	0.8747 (5)	0.037 (3)	
C18	0.4766 (4)	0.6866 (18)	0.9039 (5)	0.035 (3)	
C19	0.4594 (4)	0.4805 (19)	0.8945 (5)	0.039 (3)	
H19	0.4697	0.3999	0.8702	0.046*	
C20	0.4277 (5)	0.383 (2)	0.9188 (5)	0.047 (3)	
H20	0.4145	0.2439	0.9091	0.056*	
C21	0.4159 (5)	0.496 (2)	0.9581 (5)	0.049 (4)	
C22	0.4335 (5)	0.707 (2)	0.9716 (6)	0.055 (4)	
H22	0.4253	0.7816	0.9982	0.066*	
C23	0.4636 (5)	0.802 (2)	0.9445 (5)	0.044 (3)	
H23	0.4755	0.9436	0.9529	0.053*	
C24	0.9374 (4)	0.761 (2)	0.6852 (5)	0.038 (3)	
H24	0.9624	0.8706	0.7080	0.046*	
C25	0.8896 (4)	0.8805 (19)	0.6407 (5)	0.038 (3)	
C26	0.8384 (5)	0.781 (2)	0.6185 (6)	0.054 (4)	
H26	0.8325	0.6467	0.6310	0.065*	
C27	0.7960 (6)	0.888 (3)	0.5768 (6)	0.066 (4)	
H27	0.7617	0.8256	0.5618	0.079*	
C28	0.8050 (6)	1.079 (3)	0.5588 (6)	0.064 (4)	
H28	0.7770	1.1436	0.5298	0.076*	
C29	0.8540 (7)	1.186 (2)	0.5814 (7)	0.071 (5)	
H29	0.8589	1.3230	0.5698	0.085*	
C30	0.8965 (6)	1.0765 (19)	0.6230 (6)	0.051 (3)	
H30	0.9304	1.1429	0.6386	0.062*	
C31	0.9696 (4)	0.6227 (19)	0.6584 (5)	0.039 (3)	
H31A	0.9914	0.5190	0.6853	0.047*	
H31B	0.9436	0.5421	0.6284	0.047*	
C32	1.0069 (5)	0.742 (2)	0.6365 (5)	0.044 (3)	
C33	1.0324 (4)	0.624 (2)	0.6030 (5)	0.040 (3)	
C34	1.0175 (5)	0.418 (2)	0.5843 (5)	0.045 (3)	
H34	0.9897	0.3489	0.5915	0.054*	
C35	1.0436 (5)	0.314 (2)	0.5549 (6)	0.053 (4)	
H35	1.0339	0.1731	0.5430	0.064*	
C36	1.0838 (5)	0.415 (2)	0.5429 (5)	0.047 (3)	
C37	1.1004 (5)	0.618 (3)	0.5617 (5)	0.056 (4)	
H37	1.1283	0.6854	0.5544	0.067*	
C38	1.0745 (5)	0.719 (2)	0.5920 (5)	0.049 (3)	
H38	1.0859	0.8565	0.6055	0.058*	
C39	0.9204 (5)	0.617 (2)	0.7231 (5)	0.043 (3)	
H39A	0.8965	0.5048	0.7014	0.051*	
H39B	0.9526	0.5479	0.7491	0.051*	
C40	0.8916 (4)	0.738 (2)	0.7544 (5)	0.043 (3)	
C41	0.8558 (4)	0.6114 (19)	0.7764 (5)	0.039 (3)	
C42	0.8393 (5)	0.403 (2)	0.7599 (5)	0.046 (3)	
H42	0.8515	0.3319	0.7356	0.055*	
C43	0.8043 (5)	0.301 (2)	0.7804 (5)	0.047 (3)	
H43	0.7928	0.1610	0.7694	0.057*	
C44	0.7867 (5)	0.405 (2)	0.8165 (6)	0.049 (3)	
C45	0.8037 (6)	0.611 (2)	0.8350 (6)	0.059 (4)	
H45	0.7932	0.6777	0.8611	0.071*	
C46	0.8373 (5)	0.713 (2)	0.8129 (5)	0.044 (3)	
H46	0.8477	0.8550	0.8229	0.053*	

### Atomic displacement parameters (Å^2^)

**Table:**

	*U*^11^	*U*^22^	*U*^33^	*U*^12^	*U*^13^	*U*^23^
Br1	0.0720 (11)	0.0805 (12)	0.0863 (12)	−0.0038 (9)	0.0490 (10)	−0.0211 (9)
Br2	0.0631 (10)	0.1033 (14)	0.0771 (12)	−0.0008 (9)	0.0424 (9)	0.0248 (10)
Br3	0.0795 (11)	0.1037 (14)	0.0675 (11)	0.0286 (10)	0.0453 (9)	−0.0057 (9)
Br4	0.0786 (11)	0.0765 (12)	0.1146 (15)	−0.0054 (9)	0.0681 (11)	0.0137 (10)
O1	0.076 (7)	0.046 (6)	0.070 (7)	−0.008 (5)	0.052 (6)	−0.014 (5)
O2	0.061 (6)	0.028 (6)	0.115 (9)	0.010 (4)	0.041 (6)	−0.004 (5)
O3	0.059 (6)	0.050 (6)	0.070 (6)	−0.014 (4)	0.043 (5)	−0.010 (5)
O4	0.081 (7)	0.047 (6)	0.069 (7)	−0.013 (5)	0.045 (6)	−0.005 (5)
C1	0.031 (6)	0.035 (7)	0.064 (9)	−0.008 (5)	0.023 (6)	−0.016 (6)
C2	0.041 (7)	0.030 (7)	0.056 (8)	0.000 (5)	0.028 (6)	−0.001 (6)
C3	0.043 (8)	0.047 (9)	0.067 (10)	0.001 (6)	0.023 (7)	−0.008 (7)
C4	0.065 (10)	0.052 (10)	0.070 (11)	−0.022 (8)	0.028 (9)	−0.014 (8)
C5	0.049 (9)	0.077 (12)	0.050 (9)	−0.013 (8)	0.008 (7)	−0.004 (8)
C6	0.057 (9)	0.053 (10)	0.082 (11)	−0.002 (7)	0.029 (9)	0.013 (9)
C7	0.048 (8)	0.040 (8)	0.055 (9)	−0.007 (6)	0.025 (7)	−0.002 (6)
C8	0.070 (9)	0.040 (8)	0.042 (8)	−0.010 (6)	0.020 (7)	−0.001 (6)
C9	0.049 (8)	0.037 (9)	0.038 (7)	−0.003 (6)	0.018 (6)	0.001 (6)
C10	0.040 (7)	0.040 (8)	0.044 (8)	−0.011 (5)	0.013 (6)	−0.009 (6)
C11	0.044 (7)	0.035 (8)	0.064 (9)	−0.004 (5)	0.025 (7)	0.008 (6)
C12	0.056 (8)	0.060 (10)	0.059 (9)	0.003 (7)	0.035 (7)	0.012 (7)
C13	0.041 (7)	0.044 (8)	0.052 (8)	−0.005 (6)	0.021 (6)	−0.003 (6)
C14	0.057 (8)	0.044 (8)	0.043 (8)	−0.006 (6)	0.023 (7)	−0.004 (6)
C15	0.049 (7)	0.030 (7)	0.045 (8)	−0.017 (6)	0.019 (6)	0.002 (6)
C16	0.042 (7)	0.040 (8)	0.051 (8)	0.003 (5)	0.024 (6)	0.006 (6)
C17	0.027 (6)	0.034 (8)	0.040 (7)	0.001 (5)	0.001 (5)	−0.003 (5)
C18	0.030 (6)	0.038 (8)	0.034 (7)	0.004 (5)	0.010 (5)	−0.001 (5)
C19	0.036 (7)	0.035 (8)	0.045 (8)	−0.005 (5)	0.015 (6)	−0.012 (6)
C20	0.055 (8)	0.040 (8)	0.043 (8)	−0.014 (6)	0.016 (7)	−0.004 (6)
C21	0.030 (7)	0.072 (10)	0.046 (8)	0.001 (6)	0.016 (6)	0.018 (7)
C22	0.061 (9)	0.056 (10)	0.058 (9)	−0.002 (7)	0.035 (8)	−0.022 (7)
C23	0.045 (7)	0.048 (8)	0.042 (7)	−0.012 (6)	0.019 (6)	−0.014 (6)
C24	0.025 (6)	0.039 (7)	0.053 (8)	−0.001 (5)	0.018 (6)	−0.003 (6)
C25	0.032 (6)	0.042 (8)	0.049 (8)	−0.002 (5)	0.026 (6)	0.002 (6)
C26	0.044 (8)	0.052 (9)	0.062 (9)	0.003 (6)	0.017 (7)	0.005 (7)
C27	0.045 (8)	0.097 (14)	0.047 (9)	0.010 (8)	0.007 (7)	−0.017 (9)
C28	0.059 (10)	0.063 (11)	0.070 (11)	0.042 (8)	0.025 (9)	0.026 (9)
C29	0.097 (13)	0.040 (9)	0.084 (12)	0.025 (9)	0.044 (11)	0.010 (8)
C30	0.061 (9)	0.025 (7)	0.069 (10)	0.008 (6)	0.025 (8)	0.010 (6)
C31	0.031 (6)	0.037 (7)	0.049 (8)	−0.001 (5)	0.016 (6)	0.007 (6)
C32	0.050 (8)	0.033 (8)	0.046 (8)	−0.020 (6)	0.015 (6)	−0.012 (6)
C33	0.031 (6)	0.042 (8)	0.048 (8)	0.003 (5)	0.016 (6)	0.013 (6)
C34	0.048 (7)	0.042 (8)	0.059 (9)	0.010 (6)	0.035 (7)	0.001 (6)
C35	0.060 (9)	0.047 (9)	0.058 (9)	0.011 (7)	0.029 (7)	0.004 (7)
C36	0.043 (7)	0.065 (10)	0.040 (8)	0.021 (7)	0.024 (6)	0.003 (7)
C37	0.045 (8)	0.083 (11)	0.052 (9)	−0.015 (7)	0.032 (7)	−0.019 (8)
C38	0.041 (7)	0.056 (9)	0.055 (8)	−0.017 (6)	0.024 (7)	−0.009 (7)
C39	0.042 (7)	0.047 (8)	0.049 (8)	0.011 (6)	0.030 (6)	0.011 (6)
C40	0.029 (6)	0.056 (10)	0.047 (8)	0.011 (6)	0.016 (6)	0.018 (7)
C41	0.035 (6)	0.032 (7)	0.062 (8)	0.005 (5)	0.031 (6)	0.005 (6)
C42	0.049 (8)	0.043 (8)	0.049 (8)	0.006 (6)	0.024 (7)	0.000 (6)
C43	0.052 (8)	0.040 (8)	0.057 (9)	−0.006 (6)	0.029 (7)	−0.003 (6)
C44	0.045 (7)	0.046 (9)	0.063 (9)	0.007 (6)	0.028 (7)	0.024 (7)
C45	0.069 (9)	0.055 (10)	0.067 (10)	0.011 (7)	0.043 (8)	0.013 (8)
C46	0.056 (8)	0.031 (8)	0.046 (8)	−0.002 (6)	0.021 (7)	−0.007 (6)

### Geometric parameters (Å, °)

**Table:**

Br1—C13	1.889 (12)	C21—C22	1.387 (19)
Br2—C21	1.900 (11)	C22—C23	1.386 (17)
Br3—C36	1.902 (11)	C22—H22	0.9300
Br4—C44	1.914 (11)	C23—H23	0.9300
O1—C9	1.240 (14)	C24—C39	1.526 (15)
O2—C17	1.156 (13)	C24—C31	1.556 (15)
O3—C32	1.240 (14)	C24—C25	1.553 (16)
O4—C40	1.223 (15)	C24—H24	0.9800
C1—C2	1.494 (17)	C25—C30	1.340 (16)
C1—C16	1.536 (15)	C25—C26	1.403 (17)
C1—C8	1.561 (16)	C26—C27	1.41 (2)
C1—H1	0.9800	C26—H26	0.9300
C2—C3	1.385 (17)	C27—C28	1.33 (2)
C2—C7	1.427 (17)	C27—H27	0.9300
C3—C4	1.420 (19)	C28—C29	1.38 (2)
C3—H3	0.9300	C28—H28	0.9300
C4—C5	1.37 (2)	C29—C30	1.42 (2)
C4—H4	0.9300	C29—H29	0.9300
C5—C6	1.36 (2)	C30—H30	0.9300
C5—H5	0.9300	C31—C32	1.511 (15)
C6—C7	1.37 (2)	C31—H31A	0.9700
C6—H6	0.9300	C31—H31B	0.9700
C7—H7	0.9300	C32—C33	1.488 (16)
C8—C9	1.481 (16)	C33—C34	1.375 (17)
C8—H8A	0.9700	C33—C38	1.384 (15)
C8—H8B	0.9700	C34—C35	1.377 (16)
C9—C10	1.482 (16)	C34—H34	0.9300
C10—C11	1.398 (15)	C35—C36	1.370 (17)
C10—C15	1.403 (16)	C35—H35	0.9300
C11—C12	1.418 (17)	C36—C37	1.363 (19)
C11—H11	0.9300	C37—C38	1.382 (16)
C12—C13	1.384 (18)	C37—H37	0.9300
C12—H12	0.9300	C38—H38	0.9300
C13—C14	1.371 (16)	C39—C40	1.516 (16)
C14—C15	1.389 (16)	C39—H39A	0.9700
C14—H14	0.9300	C39—H39B	0.9700
C15—H15	0.9300	C40—C41	1.503 (15)
C16—C17	1.527 (16)	C41—C42	1.384 (17)
C16—H16A	0.9700	C41—C46	1.385 (15)
C16—H16B	0.9700	C42—C43	1.384 (16)
C17—C18	1.514 (16)	C42—H42	0.9300
C18—C19	1.349 (16)	C43—C44	1.369 (17)
C18—C23	1.434 (15)	C43—H43	0.9300
C19—C20	1.372 (16)	C44—C45	1.38 (2)
C19—H19	0.9300	C45—C46	1.383 (17)
C20—C21	1.381 (17)	C45—H45	0.9300
C20—H20	0.9300	C46—H46	0.9300
			
C2—C1—C16	111.9 (11)	C39—C24—C31	108.5 (9)
C2—C1—C8	110.8 (9)	C39—C24—C25	114.6 (8)
C16—C1—C8	109.1 (9)	C31—C24—C25	110.6 (9)
C2—C1—H1	108.3	C39—C24—H24	107.6
C16—C1—H1	108.3	C31—C24—H24	107.6
C8—C1—H1	108.3	C25—C24—H24	107.6
C3—C2—C7	116.0 (12)	C30—C25—C26	119.5 (12)
C3—C2—C1	121.5 (11)	C30—C25—C24	121.1 (11)
C7—C2—C1	122.5 (11)	C26—C25—C24	119.4 (11)
C2—C3—C4	121.9 (13)	C25—C26—C27	118.6 (14)
C2—C3—H3	119.0	C25—C26—H26	120.7
C4—C3—H3	119.0	C27—C26—H26	120.7
C5—C4—C3	119.5 (14)	C28—C27—C26	120.2 (15)
C5—C4—H4	120.3	C28—C27—H27	119.9
C3—C4—H4	120.3	C26—C27—H27	119.9
C4—C5—C6	119.6 (14)	C27—C28—C29	122.7 (14)
C4—C5—H5	120.2	C27—C28—H28	118.7
C6—C5—H5	120.2	C29—C28—H28	118.7
C7—C6—C5	121.7 (14)	C28—C29—C30	116.8 (14)
C7—C6—H6	119.1	C28—C29—H29	121.6
C5—C6—H6	119.1	C30—C29—H29	121.6
C6—C7—C2	121.1 (13)	C25—C30—C29	122.2 (14)
C6—C7—H7	119.4	C25—C30—H30	118.9
C2—C7—H7	119.4	C29—C30—H30	118.9
C9—C8—C1	113.7 (10)	C32—C31—C24	116.7 (10)
C9—C8—H8A	108.8	C32—C31—H31A	108.1
C1—C8—H8A	108.8	C24—C31—H31A	108.1
C9—C8—H8B	108.8	C32—C31—H31B	108.1
C1—C8—H8B	108.8	C24—C31—H31B	108.1
H8A—C8—H8B	107.7	H31A—C31—H31B	107.3
O1—C9—C8	119.1 (11)	O3—C32—C33	120.5 (11)
O1—C9—C10	120.2 (10)	O3—C32—C31	120.2 (11)
C8—C9—C10	120.3 (11)	C33—C32—C31	119.3 (11)
C11—C10—C15	119.5 (11)	C34—C33—C38	117.8 (11)
C11—C10—C9	118.6 (11)	C34—C33—C32	122.6 (10)
C15—C10—C9	121.9 (10)	C38—C33—C32	119.5 (11)
C10—C11—C12	119.2 (12)	C33—C34—C35	120.2 (11)
C10—C11—H11	120.4	C33—C34—H34	119.9
C12—C11—H11	120.4	C35—C34—H34	119.9
C13—C12—C11	119.1 (11)	C36—C35—C34	120.6 (13)
C13—C12—H12	120.4	C36—C35—H35	119.7
C11—C12—H12	120.4	C34—C35—H35	119.7
C14—C13—C12	122.1 (12)	C37—C36—C35	120.9 (11)
C14—C13—Br1	118.3 (10)	C37—C36—Br3	120.4 (9)
C12—C13—Br1	119.4 (9)	C35—C36—Br3	118.7 (11)
C13—C14—C15	119.0 (12)	C36—C37—C38	117.9 (11)
C13—C14—H14	120.5	C36—C37—H37	121.1
C15—C14—H14	120.5	C38—C37—H37	121.1
C14—C15—C10	120.8 (11)	C37—C38—C33	122.6 (13)
C14—C15—H15	119.6	C37—C38—H38	118.7
C10—C15—H15	119.6	C33—C38—H38	118.7
C17—C16—C1	111.7 (10)	C40—C39—C24	113.4 (10)
C17—C16—H16A	109.3	C40—C39—H39A	108.9
C1—C16—H16A	109.3	C24—C39—H39A	108.9
C17—C16—H16B	109.3	C40—C39—H39B	108.9
C1—C16—H16B	109.3	C24—C39—H39B	108.9
H16A—C16—H16B	107.9	H39A—C39—H39B	107.7
O2—C17—C18	121.2 (11)	O4—C40—C41	121.7 (11)
O2—C17—C16	123.5 (11)	O4—C40—C39	120.1 (11)
C18—C17—C16	115.2 (10)	C41—C40—C39	118.1 (12)
C19—C18—C23	116.9 (10)	C42—C41—C46	119.6 (10)
C19—C18—C17	127.3 (10)	C42—C41—C40	122.5 (11)
C23—C18—C17	115.8 (10)	C46—C41—C40	117.8 (11)
C18—C19—C20	123.9 (11)	C41—C42—C43	118.9 (11)
C18—C19—H19	118.1	C41—C42—H42	120.5
C20—C19—H19	118.1	C43—C42—H42	120.5
C21—C20—C19	118.2 (12)	C44—C43—C42	120.4 (12)
C21—C20—H20	120.9	C44—C43—H43	119.8
C19—C20—H20	120.9	C42—C43—H43	119.8
C22—C21—C20	121.7 (11)	C43—C44—C45	122.0 (11)
C22—C21—Br2	119.0 (10)	C43—C44—Br4	118.4 (10)
C20—C21—Br2	119.3 (10)	C45—C44—Br4	119.4 (10)
C21—C22—C23	118.2 (11)	C46—C45—C44	117.0 (12)
C21—C22—H22	120.9	C46—C45—H45	121.5
C23—C22—H22	120.9	C44—C45—H45	121.5
C22—C23—C18	121.0 (11)	C45—C46—C41	122.1 (12)
C22—C23—H23	119.5	C45—C46—H46	119.0
C18—C23—H23	119.5	C41—C46—H46	119.0
